# Analysis of Risk Factors for the Recurrence of Chronic Tic Disorder in Children

**DOI:** 10.1111/jpc.70170

**Published:** 2025-08-24

**Authors:** Miao Jing, Yanping Wang, Jingbo Ma, Xiaoyue Hu, Lin Zhang, Ying Hua, Jianbiao Wang

**Affiliations:** ^1^ Department of Neurology The Affiliated Wuxi Children's Hospital of JiangNan University Wuxi Jiangsu Province China

**Keywords:** aripiprazole, chronic tic disorders in children, *Mycoplasma pneumoniae* antibody IgM, recurrence risk factors, vocal tics

## Abstract

**Objective:**

To identify risk factors for recurrence in children with chronic tic disorders after 1 year of medication treatment and 6 months post‐medication discontinuation.

**Methods:**

A two‐phase hybrid design study was conducted at Wuxi Children's Hospital, involving 124 paediatric patients with chronic tic disorders treated between January 2020 and December 2022. After 1 year of medication treatment, patients were categorised into relapse and no relapse groups based on recurrence within 6 months post‐medication discontinuation. Clinical data from both groups were compared, and multivariate binary logistic regression analysis was used to determine independent risk factors for recurrence.

**Results:**

Significant differences were found between the relapse and no relapse groups in age (*p* = 0.003), white blood cell count (*p* = 0.001), *Mycoplasma pneumoniae* antibody IgM (*p* = 0.009), tic characteristics (*p* = 0.025), and medication treatment (*p* = 0.001). Binary logistic regression analysis identified *Mycoplasma pneumoniae* antibody IgM (OR = 4.797, 95% CI: 1.826–12.605) and vocal tics (OR = 8.202, 95% CI: 2.751–24.455) as independent risk factors for recurrence. Age (OR = 0.519, 95% CI: 0.335–0.803) and Aripiprazole (OR = 0.091, 95% CI: 0.031–0.271) were identified as protective factors.

**Conclusion:**

Mycoplasma infection, and vocal tics are significant risk factors for recurrence in children with chronic tic disorders. Increased age and the use of aripiprazole may serve as protective factors and be considered for clinical management of chronic tic disorders in children.

Childhood tic disorder is a common childhood‐onset neurological disorder characterised primarily by tics. Currently, the reported annual incidence rate of tic disorder in China is 6.1% [1, 2], with the peak onset age ranging from 2 to 15 years. The aetiology of tic disorder remains unclear, but ongoing research suggests it may result from a combination of factors, including immunological, neurotransmitter abnormalities, genetic predisposition and psychological elements. One of the widely acknowledged mechanisms is the phenomenon of excessive dopamine activity or hypersensitivity of dopamine receptors. According to this theory and the 2020 Chinese expert consensus on the diagnosis and treatment of tic disorder, tiapride and aripiprazole are recommended as first‐line therapeutic medications. For children with tic disorder whose tics significantly impact daily life and learning, pharmacological treatment is warranted when psychological education and behavioural therapy prove ineffective or cannot provide adequate control [3, 4].

Children with tic disorder may exhibit motor or vocal tics and are prone to co‐occurring conditions such as attention‐deficit/hyperactivity disorder (ADHD), oppositional defiant disorder (ODD) and obsessive‐compulsive disorder (OCD). It has become a common neurodevelopmental disorder in childhood. The symptoms are complex, and the course of the disorder is often prolonged with a tendency for recurrence, posing a certain economic burden on both society and families [5, 6, 7]. Therefore, early detection and intervention are crucial. However, even after achieving control over tic symptoms, the recurrence of tics remains a significant concern for both parents and clinical practitioners.

Currently, research on relapse is relatively limited, often focusing on single factors without comprehensive exploration from baseline, clinical phenotypes, infectious factors, tic types, etc. There is currently no clear and unified standard. This study, through a retrospective analysis of 124 clinical cases at Wuxi Children's Hospital, reviews general information about children, infectious factors, tic characteristics and medication treatment. The aim is to explore the independent risk factors for relapse in children with chronic tic disorders (CTDs), providing some theoretical support for the diagnosis and treatment of tic disorders, and offering partial data reference for clinical efforts in relapse prevention.

## 1 Object and Methods

### 1.1 Study Population

This study utilised a two‐phase hybrid design to investigate relapse predictors in CTD. Prospective randomised controlled trial (RCT) phase: children with CTD (diagnosed per DSM‐5 criteria as motor or vocal tics persisting ≥ 1 year, excluding Tourette syndrome [TS] with both tic types) and a Yale Global Tic Severity Scale (YGTSS) score > 25 were randomly assigned to aripiprazole or tiapride using a computer‐generated randomisation sequence. The duration of tic symptoms prior to enrolment was confirmed through medical records and caregiver reports, with a minimum requirement of 12 months and no exclusion based on an upper limit (range: 12 months to 8 years; mean ± SD: 3.2 ± 1.8 years; median: 2.5 years). After 1 year of standardised treatment and subsequent medication discontinuation, patients were retrospectively categorised into relapse (symptom recurrence) and no relapse (symptom‐free) groups based on 6‐month follow‐up outcomes. The no relapse group comprised children who did not exhibit symptoms 6 months post‐discontinuation. This study was conducted in accordance with the ethical standards of the institutional review board (IRB) of Wuxi Children's Hospital. Ethical approval was granted under protocol number (WXCH2023‐02‐042). Prior to participation, all guardians of the children were informed about the study, and written informed consent was obtained from each guardian by signing a consent form.

### 1.2 Inclusion and Exclusion

#### 1.2.1 Common Inclusion Criteria for all Participants

1. Diagnosis of CTD with symptom duration ≥ 1 year at enrollment;
2. Age 6–16 years;
3. YGTSS total score > 25 (moderate‐to‐severe tics defined as ≥ 25 based on the 2020 Paediatric Tourette's Disorder Consensus Guidelines).

#### 1.2.2 Additional Criteria Inclusion Criteria for the Relapse Group

(1) Recurrence 6 months after 1 year of standard treatment. (2) Complete clinical data. Inclusion criteria for the no relapse group: (1) No symptoms 6 months post‐treatment. (2) Outpatient follow‐up without external institution visits. (3) Complete clinical data.

Exclusion criteria for both groups: (1) No medication treatment at the outpatient clinic. (2) Missing data or family withdrawal.

### 1.3 Methods

#### 1.3.1 Data Collection

Clinical data collection included gender, age, visitation time, tic types (motor, vocal), family history (siblings, parental mental health), sleep disorders, lab results (white blood cell count, anti‐streptolysin O, *Mycoplasma pneumoniae* IgM), medication history (aripiprazole, tiapride), YGTSS scores (before and after treatment, after recurrence) and treatment course. Follow‐up included telephone or outpatient visits, with clinic details documented.

#### 1.3.2 Classification of Tourette's Disorder (TD)

Tourette's disorder (TD) was classified into three types based on clinical features and illness duration: Transient tic disorder, CTD and TS. This study included only children with CTD, presenting either motor or vocal tics. Diagnostic criteria followed the ‘Expert Consensus on the Diagnosis and Treatment of Pediatric Tourette's Disorder (2020 Edition)’.

YGTSS assessment severity was quantified using the YGTSS total score (range 0–100), comprising: tic severity subscale (motor + vocal tics: 0–25 each); Impairment subscale (0–50). ‘Moderate‐to‐severe’ was defined as total score ≥ 25 per consensus guidelines [8]. Scores were assessed at baseline, treatment end and 6‐month follow‐up.

#### 1.3.3 Remission and Relapse Definitions

Remission: YGTSS total score ≤ 4 (no functional impairment) and clinician‐confirmed absence of observable tics for ≥ 6 months.

Relapse: YGTSS ≥ 5 or recurrence of ≥ 1 tic causing functional/psychological distress [9, 10].

#### 1.3.4 Treatment Methods

Tiapride (Jiangsu Enhua Pharmaceutical Co. Ltd., 100 mg/tablet): initial dose of 50–100 mg/day, administered two to three times a day. Dosage was adjusted every 1–2 weeks based on the patient's condition, up to 200–400 mg/day. Aripiprazole (Zhejiang Otsuka Pharmaceutical Co. Ltd., 5 mg/tablet): initial dose of 1.25 mg/day, increased to 2.5 mg/day after 2 days, then adjusted to 5 mg/day within 2–5 days, further adjusted to 5–15 mg/day depending on the patient's condition. All enrolled children received routine psychoeducation about tic disorders, but no standardised psychological intervention protocols (e.g., CBIT) were implemented.

#### 1.3.5 Observation Indices

A follow‐up for 124 children with CTD was conducted through clinic visits or telephone inquiries. Data collected included medication usage (collecting 2 mL fasting venous blood before and after treatment, centrifuging the serum, monitoring blood routine, *Mycoplasma pneumoniae* antibodies, anti‐streptolysin O and electrocardiogram) and symptom relief. Recurrence was assessed (Yes, No).

### 1.4 Statistical Methods

SPSS 26.0 was used for data analysis, with significance set at *p* < 0.05. Descriptive statistics for continuous variables were presented as mean ± standard deviation. *t*‐tests were used for continuous variables, while categorical variables were analysed using the chi‐square test. Significant and clinically relevant variables were included in a multifactorial binary logistic regression model to explore relationships between multiple factors and tic disorder relapse.

## 2 Results

### 2.1 Baseline Data

There were 57 cases (46.0%) in the relapse group, with 31 boys and 26 girls. The age of onset ranged from 5 to 12 years, with a mean age of (8.73 ± 1.35) years. In the no relapse group, there were 67 cases (54.0%), with 37 boys and 30 girls. The onset age ranged from 5 to 13 years, with a mean age of (8.48 ± 1.42) years. Compared to the no relapse group, the relapse group showed significant differences in age, white blood cell count, *Mycoplasma pneumoniae* antibodies IgM, tic symptoms, and medication treatment (all *p* < 0.05) (see Table 1).

**TABLE 1:** Comparison of baseline data of two groups of children.

Item	Recurrence group (*n* = 57)	No relapse group (*n* = 67)	*t*/*χ* ^2^	*p*
General characteristics				
Age (years)	8.73 ± 1.35	8.48 ± 1.42	−3.076	0.003
Male sex	31 (54.4%)	37 (55.2%)	0.009	0.926
Family structure				
Has siblings (yes)	23 (40.4%)	31 (46.3%)	0.439	0.508
Comorbidities in children				
ADHD	12 (21.1%)	15 (22.4%)	0.032	0.858
OCD	8 (14.0%)	10 (14.9%)	0.018	0.894
Sleep disorders	26 (45.6%)	32 (47.8%)	0.057	0.811
Parental mental disorders				
Depression	14 (24.6%)	12 (17.9%)	0.782	0.377
Anxiety disorders	11 (19.3%)	11 (16.4%)	0.166	0.684
Auxiliary examination				
White blood cells (×10^9^/L)	6.59 ± 1.49	7.76 ± 1.76	−3.574	0.001
Anti‐streptolysin O (IU/mL)	186.56 ± 120.09	180.20 ± 62.10	0.49	0.626
Mycoplasma antibody IgM(+)	38 (66.7%)	29 (43.3%)	6.78	0.009
Tic characteristics				
Motor tic	20 (35.1%)	37 (55.2%)	5.028	0.025
Vocal tic	37 (64.9%)	30 (44.8%)	4.962	0.026
Medication history				
Tiapride	47 (82.5%)	31 (46.3%)	17.284	< 0.001
Aripiprazole	10 (17.5%)	36 (53.7%)	16.892	< 0.001

*Note: p* < 0.05, indicates statistical significance between groups.

Abbreviations: ADHD, attention‐deficit/hyperactivity disorder; OCD, obsessive‐compulsive disorder; ASO, anti‐streptolysin O.

### 2.2 Risk Factor Analysis

**FIGURE 1:**
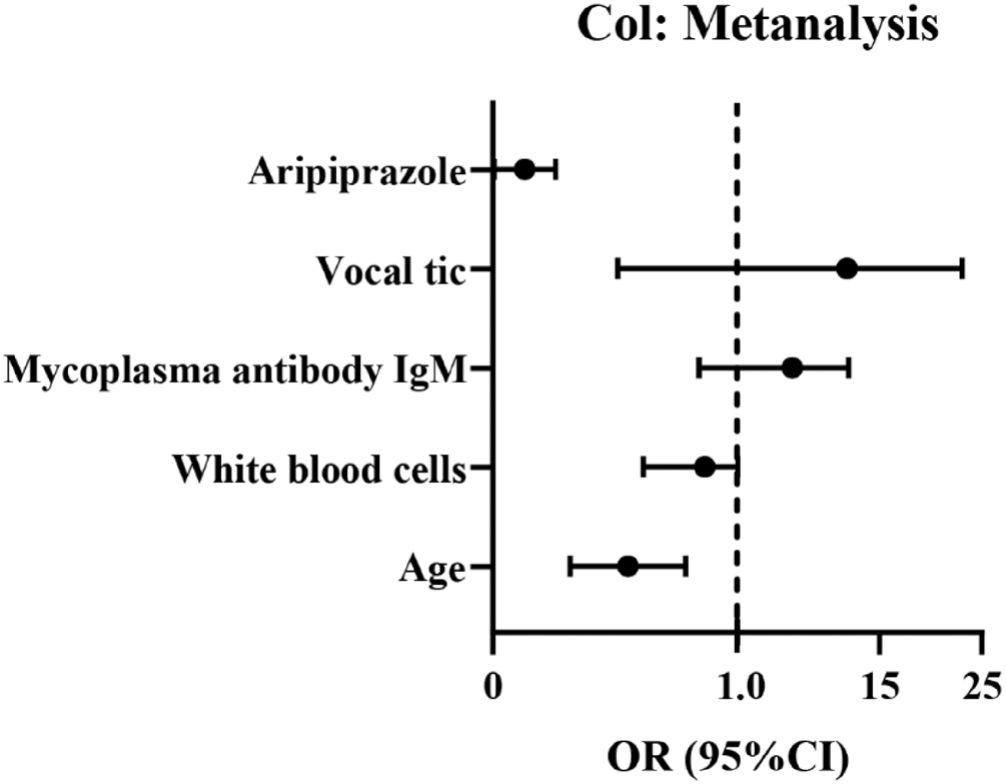
Forest plot of multifactorial logistic regression analysis of factors affecting the recurrence of chronic tic disorder in children.

Regression analysis of risk factors affecting the recurrence of tic disorders in children: in this study, factors with significant differences in the above analysis and were clinically relevant, including age, white blood cell count, *Mycoplasma pneumoniae* antibodies IgM, tic symptoms and medication treatment. These factors were considered as independent variables. The recurrence of tic disorders in children was set as the dependent variable and introduced into a multifactorial binary logistic regression equation for regression analysis. The results showed that *Mycoplasma pneumoniae* antibody IgM (OR = 4.797, 95% CI: 1.826–12.605) and vocal tics (OR = 8.202, 95% CI: 2.751–24.455) as independent risk factors for recurrence. Age (OR = 0.519, 95% CI: 0.335–0.803) and Aripiprazole (OR = 0.091, 95% CI: 0.031–0.271) were identified as a protective factor (see Table 2, Figure 1).

**TABLE 2:** Multifactorial logistic regression analysis of factors affecting recurrence of children with chronic tic disorder.

Indicator	*B*	SE	Walds	*p*	OR	95% CI
Age	−0.656	0.223	8.684	0.003	0.519	0.335–0.803
White blood cells	−0.173	0.149	1.359	0.244	0.841	0.629–1.125
Mycoplasma antibody IgM	1.568	0.493	10.121	0.001	4.797	1.826–12.605
Vocal tic	2.104	0.557	14.251	0.001	8.202	2.751–24.455
Aripiprazole	−2.359	0.556	18.583	0.001	0.091	0.031–0.271
Constant	5.698	1.871	9.276	0.002	298.200	

## 3 Discussion

Tic disorder is a neurodevelopmental disorder characterised by involuntary, repetitive, rapid and purposeless motor and/or vocal tics, commonly observed in childhood. This condition significantly impacts a child's learning, social abilities and personality development. Therefore, early and appropriate pharmacological intervention is crucial, especially for children with moderate to severe CTDs. In clinical practice, we have observed a relatively high recurrence rate after discontinuation of medication, leading to increased negative emotions for both the affected children and their parents. Hence, analysing the risk factors for recurrence after discontinuation is crucial for reducing the re‐visit rate, alleviating anxiety for both parents and children, and enhancing the child's confidence.

The data from this study demonstrate that the recurrence group had significantly higher white blood cell (WBC) counts compared to the no relapse group (Table 1: *p* = 0.001), suggesting a phenotypic association with tic relapse that may reflect short‐term immune activation. However, after adjusting for age, sex, infection history and medication use, the independent predictive effect of WBC disappeared (Table 2: *p* = 0.244, OR = 0.841, 95% CI 0.629–1.125). This discrepancy implies that elevated WBC levels may be a concomitant phenomenon of confounding factors (e.g., recent infections) or require indirect effects via downstream inflammatory mediators (e.g., CRP, IL‐6) to disrupt neurotransmitter balance, rather than acting as a direct pathogenic driver. The inconsistency with findings from Kim B et al. may stem from differences in study populations or endpoint definitions (e.g., this study focused on 6‐month relapse rather than acute exacerbation). These results align with the complexity of ‘neuroimmune interactions’, where WBC serves as an acute‐phase biomarker, but chronic relapse likely involves multifactorial synergism rather than independent prediction by a single indicator [11]. Interestingly, age emerged as a protective factor in the binary logistic regression analysis (Table 2: *p* = 0.03, OR = 0.519, 95% CI: 0.335–0.803). These data indicate that each 1‐year increase in age reduces the risk of tic relapse by 40.9%, suggesting that advancing age may confer a protective effect. Epidemiological evidence shows that tic symptoms predominantly manifest before age 18 (typically occurring between ages 4 and 8, with a mean onset age < 7 years). This phenomenon may be attributed to improved emotional stability, enhanced problem‐solving abilities, and stronger self‐regulation skills associated with maturation, collectively mitigating relapse susceptibility [12].

Additionally, this study identified that *Mycoplasma pneumoniae* IgM antibody positivity after discontinuation of medication was associated with an increased risk of CTD recurrence (OR = 4.80, 95% CI: 1.83–12.61), consistent with previous reports of higher Mycoplasma infection rates in children with tics compared to healthy controls [13, 14, 15]. However, the exact mechanism remains unclear. While some studies suggest that immune activation (e.g., cytokine release or cross‐reactive antibodies) may contribute to symptom exacerbation [16], our findings do not establish a direct causal relationship between Mycoplasma infection and recurrence. Notably, elevated IgM titers may reflect recent immune activation rather than a specific pathogen effect, as other infections or immune dysregulation could similarly influence tic severity [17, 18]. Future studies should include broader serological testing to differentiate pathogen‐specific effects from general immune dysfunction. For children with elevated Mycoplasma antibodies and recurrent tics, a trial of immunomodulatory therapy (e.g., antibiotics or anti‐inflammatory agents) may be considered, but further controlled studies are needed to validate this approach.

This study demonstrated that vocal tics have significantly higher recurrence risk following medication discontinuation compared to motor tics (OR = 8.202, 95% CI: 2.751–24.455), establishing them as an independent risk factor. While the precise mechanisms remain unclear, emerging evidence from animal models suggests neuroanatomical pathways and genetic factors may play more substantial roles than neurotransmitter imbalances in recurrence. The D1CT‐7 transgenic mouse model [19], which carries a tic‐associated genetic mutation, showed particular promise for predicting recurrence patterns through epigenetic modifications and anatomical abnormalities. However, direct translation to human pathophysiology requires verification. Current findings align with broader research [20, 21] indicating complex interactions between neural circuitry, genetic predisposition, and symptom expression in tic disorders. Specifically, the data suggest vocal tics may be more strongly mediated by structural and genetic factors than motor tics, though this hypothesis needs validation through human neuroimaging and genetic studies. These results highlight the need for: (1) mechanistic studies differentiating vocal and motor tic pathways, and (2) development of subtype‐specific treatment approaches accounting for these biological distinctions.

This study reveals a significant protective association between aripiprazole use and reduced recurrence risk in paediatric CTDs compared to tiapride (OR = 0.09, 95% CI: 0.031–0.271). The observed clinical difference may be explained by their distinct pharmacological mechanisms: whereas tiapride acts primarily as a selective dopamine D2 receptor antagonist, aripiprazole functions as a dopamine system modulator through its partial D2 receptor agonism combined with serotonin 5‐HT1A receptor activation and 5‐HT2A receptor antagonism. This multimodal pharmacological profile potentially enables aripiprazole to maintain more balanced neural circuit activity across different functional states. Clinical evidence from previous studies [22, 23, 24] supports aripiprazole's practical advantages, including its favourable safety profile, rapid onset of action and convenient once‐daily dosing regimen that enhances treatment adherence in paediatric populations. However, several important considerations must be emphasised regarding these findings. First, the demonstrated protective association does not establish a direct causal relationship, and the actual mechanisms underlying recurrence prevention likely involve complex interactions between multiple neurotransmitter systems beyond dopamine modulation alone. Second, while animal studies suggest dopamine plays a central role in tic pathophysiology, human evidence indicates more intricate neurobiological networks are involved. Third, individual variations in drug response highlight the need for personalised treatment approaches. These findings therefore provide clinically relevant evidence supporting aripiprazole's utility as a first‐line option for moderate‐to‐severe cases, while simultaneously underscoring the importance of further research to better understand the neurobiological basis of treatment response variability and to develop more precise therapeutic strategies for CTDs.

Although multiple studies have indicated a close association between the onset of tic disorders and streptococcal infections, in the present study, anti‐streptolysin O (ASO) did not emerge as an independent risk factor for the recurrence of CTD. We speculate that ASO might be a significant factor in the onset of tic disorders but not necessarily a risk factor for recurrence. Meanwhile, we cannot rule out the potential biases due to sample size limitations. Currently, no research has demonstrated that factors such as parental history of mental illness or motor tics would influence the recurrence of tic disorders. The limited preventive efficacy of tiapride—a selective dopamine D2/D3 receptor antagonist—contrasts with the protective effects of medications with broader mechanisms (e.g., aripiprazole), indicating that single‐target dopaminergic modulation may be insufficient for recurrence prevention. This finding suggests that when selecting medications for treating CTDs, consideration should be given to the mechanism of action and targets of the drugs to achieve optimal therapeutic outcomes.

In summary, we employed multifactorial binary logistic regression to analyse risk factors for CTDs in children after 1 year of medication therapy and 6 months of discontinuation. Key findings identified mycoplasma infection and vocal tics as significant recurrence predictors, with vocal tics demonstrating a particularly strong association (OR = 8.20) though their precise neurobiological basis requires further investigation regarding potential anatomical and genetic correlates. Notably, age (> 8 years) and aripiprazole emerged as independent protective factors (OR = 0.09), supporting its clinical utility for symptom control. While this study focused on pharmacological interventions, we recognise the importance of behavioural therapies in comprehensive tic management. Future directions will: (1) expand sample sizes with multicentre cohorts, (2) incorporate standardised assessment of prior CBIT exposure as a covariate, and (3) integrate parallel clinical and animal studies to explore synergies between pharmacological and behavioural interventions (e.g., combined aripiprazole/CBIT protocols). This multidimensional approach will better elucidate recurrence mechanisms and optimise therapeutic strategies.

## Author Contributions

M.J., Y.W., X.H., J.W., L.Z., J.M. and Y.H. participated in the design of the study, collected and analysed the data, and drafted the manuscript. M.J., Y.W., L.Z. and X.H. collected the data. Y.W., J.M. and Y.H. were responsible for analysis, analysed the data, and contributed to drafting the manuscript. All authors read and approved the final manuscript.

## Conflicts of Interest

The authors declare no conflicts of interest.

## Data Availability

The original data presented in the study are publicly available. The data that support the findings of this study are available from the corresponding author upon reasonable request.
